# Phytochemical screening and in vivo antimalarial activity of extracts from three medicinal plants used in malaria treatment in Nigeria

**DOI:** 10.1007/s00436-015-4747-x

**Published:** 2015-09-22

**Authors:** A. E. Bankole, A. A. Adekunle, A. A. Sowemimo, C. E Umebese, O Abiodun, G. O. Gbotosho

**Affiliations:** Bankole A. E., Department of Botany, Faculty of Science, University of Lagos, P. M. B. 1029 Unilag Post office Akoka-Yaba, Lagos, Nigeria; Department of Pharmacognosy, Faculty of Pharmacy, University of Lagos, Lagos, Nigeria; Department of Pharmacology and Therapeutic, College of Medicine, University of Ibadan, Ibadan, Nigeria

**Keywords:** *Markhamia tomentosa*, *Plasmodium berghei*, *Polyalthia longifolia*, *Trichilia heudelotii*

## Abstract

The use of plant to meet health-care needs has greatly increased worldwide in the recent times. The search for new plant-derived bioactive agents that can be explored for the treatment of drug-resistant malaria infection is urgently needed. Thus, we evaluated the antimalarial activity of three medicinal plants used in Nigerian folklore for the treatment of malaria infection. A modified Peter’s 4-day suppressive test was used to evaluate the antimalarial activity of the plant extracts in a mouse model of chloroquine-resistant *Plasmodium berghei* ANKA strain. Animals were treated with 250, 500, or 800 mg/kg of aqueous extract. It was observed that of all the three plants studied, *Markhamia tomentosa* showed the highest chemosuppression of parasites of 73 % followed by *Polyalthia longifolia* (53 %) at day 4. All the doses tested were well tolerated. Percentage suppression of parasite growth on day 4 post-infection ranged from 1 to 73 % in mice infected with *P. berghei* and treated with extracts when compared with chloroquine diphosphate, the standard reference drug which had a chemosuppression of 90 %. The percentage survival of mice that received extract ranged from 0 to 60 % (increased as the dose increases to 800 mg/kg). Phytochemical analysis revealed the presence of tannins, saponins, and phenolic compounds in all the three plants tested.

## Introduction

There is an increasing rate of death associated with malaria disease and over one million people are affected, majority are children below 5 years (Tabuti 2008; Akanbi et al. 2012). Like other developing countries of the world, a similar condition has been observed in Nigeria. Also, the recent resistance of malaria parasites to the synthetic drugs like chloroquine which is resistant to *Plasmodium falciparum* has call for concern (Trape 2002). Due to limited availability and affordability of orthodox medicine in many tropical countries, the majority of the populations depend on traditional medical remedies (WHO 2002; Zirihi et al. 2005), mainly from plants which has been discovered to be a rich source of new drugs. Majority of the antimalarial drugs such as quinine and artemisinin that is widely used has also been developed from plants, and some were developed chemically using plant-derived compounds as pattern (Basco et al. 1994; Chiyaka et al. 2009). The World Health Organization supports the use of medicinal plants provided they are proven to be efficacious and safe (WHO 1985). The most important of these bioactive constituents of plants are alkaloids, tannins, flavonoids, and phenolic compounds.

According to an ethnobotanical survey in Nigeria (Adebayo and Krettli 2011), some 98 species from different families are used in traditional medicine singly or in combination to treat malaria and/or fever. These plants used for malaria treatment and control represent more than half of the Nigeria medicinal species. To date, there has been no literature cited to show that many of these plants have been scientifically investigated to establish whether or not they have antimalarial properties in vivo.

**Fig. 1:**
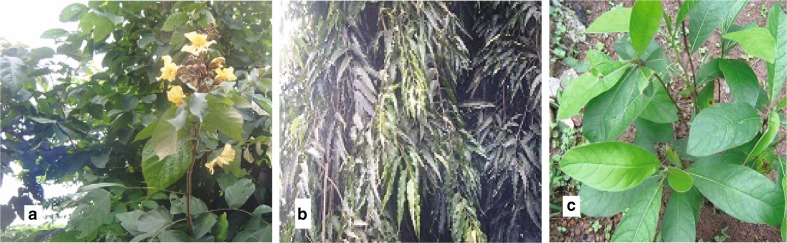
**a** *Markhamia tomentosa* plant. **b** *Polyalthia longifolia* plant. **c** *Trichilia heudelotii* plant

This paper evaluates the antimalarial activity of the extracts from three medicinal plants in the southern part of Nigeria and also investigates the qualitative and quantitative analysis of the major bioactive constituents of the three plants. The extracts were obtained from three Nigerian medicinal plants namely *Markhamia tomentosa* Benth. K. Schum. Ex Engl., *Polyalthia longifolia* Sonn. Thwaites, *Trichilia heudelotii* Planch. ex Oliv., belonging to three families, Bignoniaceae, Annonaceae, and Meliaceae, respectively. These three plants (Fig. 1) are locally used in traditional medicine to cure many diseases including malaria and fevers. (Burkill 1985; Abbiw 1990; Burkill 1997; Subramanion et al. 2013).

*M. tomentosa* is a shrub or tree of about 15-m high and is distributed in the Savannah forests in Africa. The tree possesses large yellow flowers in long terminal racemes. The leaves and stem bark are used to treat various diseases such as oedema, rheumatoid arthritis, gout, and muscular pain in Nigeria (Bouquets and Debray 1974; Burkill 1985; Arbonier 2002). Ethnopharmacological studies revealed that the leaves and stem bark of *M. tomentosa* possess antimicrobial, antiplasmodial, antioxidant, antiulcer, and analgesic properties (Aladesanmi et al. 2007; Tantangmo et al. 2010; Temdie et al. 2012; Shofidiya et al. 2014). The phytochemical screening and foliar epidermal studies of the leaves have also been carried out (Ugbabe and Ayodele 2008; Borokini and Omotayo 2012).

*P. longifolia* is an evergreen tree with a height of over 30 ft. It exhibits symmetrical pyramidal growth with willowy pendulous branches and long narrow lanceolate leaves. It originates from India and is introduced to many tropical countries around the world, Nigeria inclusive. This plant is used as an antipyretic agent in indigenous systems of medicine (Raghunathan and Mitra 1982). Ethnopharmacological studies revealed that the stem bark and leaves of this plant display effective antimicrobial activity (Faizi et al. 2003a, b, 2008), cytotoxic function (Chang et al. 2000, 2006), and hypotensive effects (Saleem et al. 2005).

*T. heudelotii* (Abbiw 1990) is a medium-sized tree up to about 20-m high and 0.4 m in girth (Irvine 1961). The plant is mostly common in the tropical rain forest in Africa. The bark is used to treat diseases such as gastrointestinal disorder, cough, gonorrhea, syphilis (Irvine 1961; Lemmens 2008), and skin ulcer (Abbiw 1990). The stem bark is also used as anthelmintic, aphrodisiac, abortifacient, and antiplasmodial (Abbiw 1990; Lemmens 2008). It also has anti-inflammatory and analgesic properties (Mshana et al. 2000; Lemmens 2008). Phytochemical screening shows the presence of tannins, saponins, glycosides, terpenes, and flavonoids.

## Materials and methods

### Plant collection and authentication

Fresh leaves of *M. tomentosa* (Benth.) K. Schum. Ex Engl. (Bignoniaceae), *P. longifolia* Sonn. Thwaites (PL). (Annonaceae) and stem bark of *T. heudelotii* Planch. ex Oliv. were collected from Oke-Igbo in Ondo State, Iddo, and Eruwa in Oyo State in Nigeria, respectively, in December 2012. Identification and authentication were carried out in the Herbarium of the Department of Botany where voucher specimens (LUH 3936, LUH 3562, and LUH 3494) were deposited.

### Plant extraction

The leaves of *M. tomentosa* and *P. longifolia* and the stem bark of *T. heudelotii* were air-dried at room temperature (30 ± 0.5 °C) and pulverized to a coarse powder. The plant parts (1 kg) were extracted exhaustively by mixing with 4 l of distilled water and allowed to boil for 4 h. The resulting extract was left to cool and filtered. The filtrates were freeze-dried, and the resulting extracts were stored at 4 °C.

### Experimental animals

Fifty-five Swiss albino mice weighing between 18–22 g of either sex and aged 6–8 weeks were kept in the Animal House of the Institute for Advanced Medical Research and Training, College of Medicine, University of Ibadan, Oyo State, Nigeria. The animals were maintained under standard laboratory conditions, as approved by the Experimentation Ethics Committee of the College of Medicine, University of Lagos (CM/COM/08/VOL.XXVI). The animals, acclimatized for 1 week, were fed on a rodent diet (Livestock Feeds PLC, Ibadan, Oyo State, Nigeria) and had free access to drinking water.

### Malaria parasite

The chloroquine-resistant *Plasmodium berghei* (ANKA) clone was used. *P. berghei* was obtained from the Malaria Research Laboratories, Institute for Advanced Medical Research and Training, College of Medicine, University of Ibadan. The experimental animals were inoculated intravenously with 1 × 10^6^ red blood cells infected with the CQ-resistant *P. berghei* ANKA strain. The day of inoculation was defined as day zero (D0). The infected animals were distributed randomly into five groups of five animals per cage and were treated once daily with the extract by oral route. The animals in group I received 250 mg/kg dose of the extract by oral route. Groups II and III received 500 and 800 mg/kg, respectively. Group IV was treated with chloroquine at a dose of 10 mg/kg. Group V served as negative control. The antimalarial activity of the extracts was determined using the Peter’s 4-day suppressive test (Peter and Anatoli 1998; David et al. 2004). Survival rate of animals were monitored till day 14. On day 4 of the experiment, the animals’ tail tip was cut to prepare the blood smears. The air-dried films were fixed with methanol and stained with Giemsa solution. Parasitemia was determined by counting the number of parasitized erythrocytes among at least 1000 RBC. In this study, in vivo antimalarial activity of the three plant extracts and the mouse survival were monitored over a period of 2 weeks

The difference between the mean values of the control group (defined as 100 %) and those of the experimental groups was calculated and expressed as percent suppression of parasite growth using the method of Li et al. (2003) and Devi et al. (2001) as shown in the equation below

$$ \mathrm{Suppression}\ \mathrm{of}\ \mathrm{parasite}=100-\frac{\mathrm{Mean}\ \mathrm{parasitaemia}\ \mathrm{of}\ \mathrm{treated}\ \mathrm{animals}}{\mathrm{Mean}\ \mathrm{parasitaemia}\ \mathrm{of}\ \mathrm{control}}\times 100 $$

### Statistical analysis

Mean parasitemia was determined in the control and treated groups. The mean parasitemia was compared using the Student’s *t* test. Percent suppression of parasite growth of the treated and control groups were compared using one-way ANOVA and two-tailed Student’s *t* test (GraphPad Prism 4.0, GraphPad Software, San Diego, USA), with *P* < 0.05 being considered significant.

### Phytochemical analysis

Chemical tests for the screening and identification of bioactive chemical constituents of the three medicinal plants were carried out in aqueous extracts using the standard procedures as described by Sofowora (1993); Trease and Evans (2002) and Harborne (1973).

#### Quantitative analysis of phytochemical constituents

##### Test for tannins

About 0.5 g of each plant extract was stirred with about 10 ml of distilled water and then filtered. Few drops of 1 % ferric chloride solution were added to 2 ml of the filtrate, and occurrence of a blue-black, green, or blue-green precipitate indicates the presence of tannins.

##### Test for steroids

In about 0.2 g of each plant extract, 2 ml of acetic acid was added, and the solution was cooled well in ice followed by the addition of concentrated H_2_SO_4_ carefully. Color development from violet to blue or bluish green indicated the presence of a steroidal ring, i.e., aglycone portion of cardiac glycoside.

##### Test for terpenoids

A little of each plant extract was dissolved in ethanol. To it, 1 ml of acetic anhydride was added followed by the addition of concentrated H_2_SO_4_. A change in color from pink to violet showed the presence of terpenoids.

##### Test for saponins

One gram of each portion was boiled with 5 ml of distilled water, filtered. To the filtrate, about 3 ml of distilled water was further added and shaken vigorously for about 5 min. Frothing which persisted on warming shows the presence of saponins.

##### Test for flavonoids

About 0.5 g of each plant extract was dissolved in ethanol, and it was warmed and then filtered. Three pieces of magnesium chips were then added to the filtrate followed by few drops of concentrated HCl. A pink, orange, or red which changed to purple color indicates the presence of flavonoids.

##### Ferric chloride test for flavonoids

About 0.5 g of each plant extract was boiled with distilled water and then filtered. To 2 ml of the filtrate, few drops of 10 % ferric chloride solution were then added. A green-blue or violet coloration indicated the presence of a phenolic hydroxyl group.

##### Glycosides

One milliliter of concentrated H_2_SO_4_ is prepared in a test tube, and 5 ml of aqueous extract from each plant sample is mixed with 2 ml of glacial CH_3_CO_2_H containing 1 drop of FeCl_3_. The above mixture is carefully added to 1 ml of concentrated H_2_SO_4_ so that the concentrated H_2_SO_4_ is underneath the mixture. A brown ring will appear indicating if cardiac glycoside is present.

##### Test for alkaloids

About 0.2 g of the each plant extract was stirred with 5 ml of 1 % aqueous HCl on water bath and then filtered. From the filtrate, 1 ml was taken individually into two test tubes. To the first portion, few drops of Dragendorff’s reagent were added; occurrence of orange-red precipitate was taken as positive. To the second 1 ml, Mayer’s reagent was added and appearance of buff-colored precipitate will be an indication for the presence of alkaloids.

##### Cardiac glycosides (Keller-Killani test)

Five milliliters of each plant extracts was treated with 2 ml of glacial acetic acid containing one drop of ferric chloride solution. One milliliter of concentrated sulphuric acid was added. A brown ring of the interface indicated a deoxysugar characteristic of cardenolides.

A violet ring may appear below the brown ring, while in the acetic acid layer, a greenish ring may form just gradually throughout a thin layer

##### Anthraquinone Borntrager’s test

About 0.5 g of the plant extract was shaken with benzene layer separated and half of its own volume of 10 % ammonia solution added. A pink, red, or violet coloration in the ammoniacal phase indicated the presence of anthraquinone.

##### Phenolic compounds

The extract (500 mg) was dissolved in 5 ml of distilled water. To this, few drops of neutral 5 % ferric chloride solution were added. A dark green color indicated the presence of phenolic compounds.

#### Quantitative determination of phytochemicals

##### Preparation of fat-free sample

Two grams of the sample were defatted with 100 ml of diethyl ether using a Soxhlet apparatus for 2 h.

##### Determination of total phenols by spectrophotometric method

The fat-free sample was boiled with 50 ml of ether for the extraction of the phenolic component for 15 min. Five milliliters of the extract was pipetted into a 50-ml flask, then 10 ml of distilled water was added. Two milliliters of ammonium hydroxide solution and 5 ml of concentrated amyl alcohol were also added. The samples were made up to mark and left to react for 30 min for color development. This was measured at 505 nm.

##### Alkaloid determination using Harborne (1973) method

Five grams of the sample was weighed into a 250-ml beaker, and 200 ml of 10 % acetic acid in ethanol was added and covered and allowed to stand for 4 h. This was filtered, and the extract was concentrated on a water bath to one quarter of the original volume. Concentrated ammonium hydroxide was added dropwise to the extract until the precipitation was complete. The whole solution was allowed to settle, and the precipitate was collected and washed with dilute ammonium hydroxide and then filtered. The residue is the alkaloid, which was dried and weighed.

##### Flavonoid determination by the method of Bohm and Kocipai-Abyazan (1994)

Ten grams of the plant sample was extracted repeatedly with 100 ml of 80 % aqueous methanol at room temperature. The whole solution was filtered through Whatman filter paper No. 42 (125 mm). The filtrate was later transferred into a crucible and evaporated into dryness over a water bath and weighed to a constant weight.

##### Saponin determination

Twenty grams of plant sample was dispersed in 200 ml of 20 % ethanol. The suspension was heated over a hot water bath for 4 h with continuous stirring at about 55 °C. The mixture was filtered and the residue re-extracted with another 200 ml of 20 % ethanol. The combined extracts were reduced to 40 ml over water bath at about 90 °C. The concentrate was transferred into a 250-ml separating funnel, and 20 ml of diethyl ether was added and shaken vigorously. The aqueous layer was recovered while the ether layer was discarded. The purification process was repeated. Sixty milliliter of normal butanol extracts were washed twice with 10 ml of 5 % aqueous sodium chloride. The remaining solution was heated in a water bath. After evaporation, the sample was dried in the oven into a constant weight. The saponin content was calculated in percentage (Nahapetian and Bassiri 1975).

## Results and discussion

The in vivo antimalarial activity of aqueous extracts of the leaves of *M. tomentosa* and *P. longifolia* and stem bark of *T. heudelotii* in chloroquine-resistant *P. berghei* (ANKA) strain was evaluated. The leaves of *P. longifolia* showed minimal suppression (53 %) of parasite multiplication at the highest dose (800 mg/kg), but the extract of *T. heudelotii* had poor suppression of parasites growth. The mean percentage parasitemia in the untreated control group ranged from 1 to 73 % on day 4 post-infections. The mice treated with chloroquine (10 mg/kg) resulted in 90 % suppression of parasite growth.

The several comparison tests indicated that the mice treated with *M. tomentosa* and *P. longifolia* extracts resulted in reduced parasite load as compared to their respective negative control groups. From the aqueous extract of *M. tomentosa* showed statistically significant (*P* < 0.05) chemosuppression against *P. berghei* in mice tested at all dose levels compared to the mice in the untreated group on day 4. In the group of animals treated with the varying doses of *T. heudelotii*, there was no suppression of parasitemia in all the days of treatment. This result however does not support the report of (Atindehou et al. 2004) that the *T. heudelotii* possesses good antimalarial activity. In the group of animals that received 800 mg/kg body weight of *M. tomentosa* and *P. longifolia*, parasite growth suppression ranged between 73 and 53 %, respectively. Moreover, the animals treated with the 800 mg/kg of *M. tomentosa* extract survived longer than the animals in the corresponding negative control groups. While in the group of animals that received 250 and 500 mg/kg of *M. tomentosa* and *P. longifolia*, parasitemia was minimally suppressed from 37 to 58 %. The animals treated with chloroquine (10 mg/kg) recorded 69 % suppression till day 7 post-infection.

In this study, remarkable suppression of parasitemia by plant extracts translated into a longer mouse survival. The survival rate of the animals after treatment with various doses of the three plant extracts was assessed. The rate of survival of the animals treated with CQ are presented in Table 1. However, the animals treated with 800 mg/kg of *M. tomentosa* and CQ showed the same survival rate of 60 % till day 14, while the untreated group did not survive till day 14. Survival time increases as the dose increases

**Table 1:** Response to treatment in Swiss albino mice infected with CQR *P. berghei* to aqueous extract of three medicinal plants at day 4

Dose of plant extracts (mg/kg)	Parasitemia ± SEM (%)	Parasite suppression ± SEM (%)	% survival till day 14
*Markhamia tomentosa* (l)			
250	6.85 ± 0.65	46	20
500	7.15 ± 0.36	43	40
800	4.96 ± 0.73	73	60
*Polyalthia longifolia* (l)			
250	12.56 ± 1.80	1	0
500	9.95 ± 0.95	21	40
800	5.94 ± 1.29	53	40
*Trichilia heudelotii* (sb)			
250	19.05 ± 4.29	0	0
500	9.95 ± 1.67	21	40
800	7.63 ± 0.47	40	40
Chloroquine (CQ)			
10	1.33 ± 0.56	90	60
Untreated control (NT)	12.66 ± 1.07		0

Values are parasite density ± standard deviation (PD ± SEM)

*CQ* Chloroquine diphosphate (10 mg/kg/day), *NT* untreated control, *l* leaf, *sb* stem bark

Larvicidal activity of *M. tomentosa* has been reported (Adebajo et al. 2012). In addition, the ethyl acetate fraction of the stem bark of *M. tomentosa* has been found to possess good in vitro antimalarial activity (Tantangmo et al. 2010).

In the qualitative and quantitative analysis of the three plants studied, some of these secondary metabolites were present and some were absent. The presence of tannins and saponins was observed in all the three plants. However, *M. tomentosa* have the highest contents of alkaloids (6.48 ± 0.18) as compared to *P. longifolia* (5.52 ± 0.18) and *T. heudelotii* (1.43 ± 0.18). The high content of alkaloids in *M. tomentosa* could be attributed to the higher antimalarial properties observed in the plant. The result in this study is similar to the result of Borokini and Omotayo (2012) who had earlier reported the presence of alkaloids in *M. tomentosa*. Alkaloids are important phytochemicals, that are said to be pharmacologically active (Trease and Evans 1989). Quinine, an alkaloid, is popular for its antimalarial activity against the malaria parasite (Iwu and Klayman 1992) (Tables 2 and 3).

**Table 2:** Qualitative phytochemical screening of the medicinal plants studied

Secondary metabolites	*Markhamia tomentosa*	*Polyalthia longifolia*	*Trichilia heudelotii*
Alkaloids	+	+	−
Tannins	+	+	+
Saponins	+	+	+
Flavonoids	+	+	−
Cardiac glycosides	−	−	+
Anthraquinones	−	−	+
Phlobatannins	−	−	+
Phenolic compounds	+	+	+
Proteins	+	+	+
Terpenes	+	−	+
Steroids	−	−	−

**Table 3:** Quantitative phytochemical screening of the medicinal plants studied

Extract	Tannins mg/100 g	Alkaloids mg/100 g	Phenols mg/100 g	Flavonoids mg/100 g	C. glycosides mg/100 g	Saponin mg/100 g	Anthraquinones mg/100 g
*Markhamia tomentosa*	29.24 ± 0.08	6.48 ± 0.18	22.73 ± 0.16	4.41 ± 0.06	0.00 ± 0.00	8.99 ± 0.23	0.00 ± 0.00
*Polyalthia longifolia*	0.00 ± 0.00	5.52 ± 0.18	0.00 ± 0.00	5.22 ± 0.19	2.65 ± 0.13	10.64 ± 0.15	0.00 ± 0.00
*Trichilia heudelotii*	46.93 ± 1.16	1.43 ± 0.18	41.41 ± 0.22	13.08 ± 0.26	3.05 ± 0.19	8.46 ± 0.23	9.57 ± 0.19

The result obtained in this study demonstrated that the aqueous leaf extract of *M. tomentosa* possesses the best potent antimalarial activity than the other two plants. This appears to be the first report of antimalarial properties of aqueous leaf extract of *M. tomentosa* in vivo*.* The result of this study support the traditional claim of the plant in the use for malaria treatment, and effort are currently underway to identify the bioactive component(s) of the plant and toxicological effects in animals.

## References

[CR1] Abbiw DK (1990). Useful plants of Ghana.

[CR2] Adebajo AC, Famuyiwa FG, John JD, Idem ES, Adeoye AO (2012). Activities of some Nigerian medicinal plants against *Aedes aegypti*. Chin Med.

[CR3] Adebayo JO, Krettli AU (2011). Potential antimalarials from Nigerian plants: a review. J Ethnopharmacol.

[CR4] Akanbi OM, Omonkhua AA, Cyril-Olutayo CA, Fasimoye RY (2012). The antiplasmodial activity of *Anogeissus leiocarpus* and its effect on oxidative stress and lipid profile in mice infected with *Plasmodium berghei*. Parasitol Res.

[CR5] Aladesanmi AJ, Iwalewa EO, Akinkunmi EO, Adebajo AC, Taiwo BJ, Olorunmola FO, Lamikanra A (2007) Antimicrobial and antioxidant activities of some nigerian medicinal plants. Afr J Tradit Complement Altern Med 4(2):173–184 10.4314/ajtcam.v4i2.31206 PMC2816440 20162089

[CR6] Arbonier M (2002). Trees, shrubs and lianas of West African dry zones.

[CR7] Atindehou KK, Schmid C, Brun R, Koné MW, Traore D (2004). Antitrypanosomal and antiplasmodial activity of medicinal plants from Côte d’Ivoire. J Ethnopharmacol.

[CR8] Basco LK, Mitaku S, Skaltsounis AL, Ravelomanaintsoa N, Tillequin F, Koch M, Le Bras J (1994). In vitro activities of acridone alkaloids against *Plasmodium falciparum*. Antimicrob Agents Chemother.

[CR9] Bohm BA, Kocipai-Abyazan R (1994). Flavonoid and condensed tannins from the leaves of *Vaccinum raticulation* and *Vaccinum calcyimium*. Pac Sci.

[CR10] Borokini TI, Omotayo FO (2012). Phytochemical and ethnobotanical study of some selected medicinal plants from Nigeria. J Med Plants Res.

[CR11] Bouquets A, Debray M (1974). Medicinal plants of the Ivory Coast. Travx Doc L’Orstom.

[CR12] Burkill HN (1985) The useful plants of west tropical Africa, Kew, published by Royal Botanic Gardens, 2nd edn. pp 258–259

[CR13] Burkill HM (1997) The useful plants of West Tropical Africa. (2nd Edition). Volume 4, Families M–R. Royal Botanic Gardens, Kew. 969 pp

[CR14] Chang FR, Hwang TL, Yang YL, Li CE, Wu CC, Issa HH (2006). Anti-inflammatory and cytotoxic diterpenes from formosan *Polyalthia longifolia* var. *pendula*. Planta Med.

[CR15] Chang FR, Shih YC, Hsieh TJ, Chia YC, Tseng HY (2000). Cytotoxic constituents of *Polyalthia longifolia* var. *pendula*. J Nat Prod.

[CR16] Chiyaka C, Garira W, Dube S (2009). Effects of treatment and drug resistance on the transmission dynamics of malaria in endemic areas. Theor Popul Biol.

[CR17] David AF, Philip JR, Simon LC, Reto B, Solomon N (2004). Antimalaria drug discovery: efficiency models for compound screening. Nat Rev.

[CR18] Devi U, Atul K, Pillai R (2001). Antiplasmodial effect of three medicinal plants: preliminary study. Curr Sci.

[CR19] Faizi S, Khan RA, Azher S, Khan SA, Tauseef S, Ahmad A (2003). New antimicrobial alkaloids from the roots of *Polyalthia longifolia* var.*pendula*. Planta Med.

[CR20] Faizi S, Khan RA, Mughal NR, Malik MS, Sajjadi KE, Ahmad A (2008). Antimicrobial activity of various parts of *Polyalthia longifolia* var.*pendula*: isolation of active principles from the leaves and the berries. Phytother Res.

[CR21] Faizi S, Mughal NR, Khan RA, Khan SA, Ahmad A, Bibi N (2003). Evaluation of the antimicrobial property of *Polyalthia longifolia* var.*pendula*: isolation of a lactone as the active antibacterial agent from the ethanol extract of the stem. Phytother Res.

[CR22] Harborne JB (1973). Phytochemical methods: a guide to modern techniques of plant analysis.

[CR23] Irvine FR (1961). Woody plants of Ghana.

[CR24] Iwu C, Klayman DL (1992). Evaluation of the in vitro antimalarial activity of *Picralima nitida* extracts. J Ethnopharmacol.

[CR25] Lemmens RH, Louppe D, Oten Amoako AA, Brink M (2008). *Trichilia monadelpha* (Thonn) J. J. de Wilde. Timbers/Bois d’oeuvre 1 [CD-Rom].

[CR26] Li G, Si Z, Lee P, Wong E, Xie H, Klye E, Dow S (2003). Efficacy of composition of intravenous artelinate and artesunate in *Plasmodium berghei*. J Ethnopharmacol.

[CR27] Mshana NR, Abbiw K, Addae-Mensah I, Adjanohoun E, Ahyi MR, Ekpere JA, Enow-Orock EG, Gbile ZO, Noamesi GK, Odei MA, Odunlami H, Oteng-Yeboah AA, Sarpong K, Soforowa A, Tackie AN (2000). Traditional medicine and pharmacopoeia. Contribution to the revision of ethnobotanical and floristic studies in Ghana.

[CR28] Nahapetian A, Bassiri A (1975). Changes in concentrations and interrelationships of phytate, phosphorus, magnesium, calcium and zinc in wheat during maturation. J Agric Food Chem.

[CR29] Peter IT, Anatoli VK (1998) The current global malaria situation. Malaria parasite biology, pathogenesis and protection. ASM Press. W.D.C., pp11-22

[CR30] Raghunathan K, Mitra R (1982) New Delhi: central council for research in Ayurveda and Siddha. Pharmacog Indigenous Drugs 127–136

[CR31] Saleem R, Ahmed M, Ahmed SI, Azeem M, Khan RA, Rasool N (2005). Hypotensive activity and toxicology of constituents from root bark of *Polyalthia longifolia* var.*pendula*. Phytother Res.

[CR32] Shofidiya MO, Agunbiade FO, Koorbanally NA, Sowemimo AA, Soesan D, Familusi T (2014). Antiulcer activity of the ethanolic extract and ethyl acetate fractions of the leaves of *Markhamia tomentosa* in rats. J Ethnopharmacol.

[CR33] Sofowora A (1993). Screening plants for bioactive agents. Medicinal plants and traditional medicinal in Africa.

[CR34] Subramanion LJ, Siew YC, Dharmaraj S, Subramanian D, Lachimanan YL, Soundararajan V, Sreenivasan S (2013). *Polyalthia longifolia* Sonn: an Ancient remedy to explore for novel therapeutic agents. Res J Pharm Biol Chem Sci.

[CR35] Tabuti JRS (2008). Herbal medicines used in the treatment of malaria in Budiope County, Uganda. J Ethnopharmacol.

[CR36] Tantangmo F, Lenta BN, Boyom FF, Ngouela S, Kaiser M, Tsamo E, Weniger B, Rosenthal PJ, Vonthron-sénécheau C (2010). Antiprotozoal activities of some constituents of *Markhamia tomentosa* (Bignoniaceae). Ann Trop Med Parasitol.

[CR37] Temdie RJG, Fotio LA, Dimo T, Beppe JG, Tsague M (2012) Analgesic and ant-inflammatory effects of the leaves of *Markhamia tomentosa* (Benth.) K. Schum. (Bignoniaceae). Pharmacologia 3:565–573

[CR38] Trape JF (2002). Combating malaria in Africa. Trends Parasitol.

[CR39] Trease GE, Evans WC (1989). Textbook of pharmacognosy.

[CR40] Trease GE, Evans WC (2002). Pharmacognosy.

[CR41] Ugbabe GE, Ayodele AE (2008). Foliar epidermal studies in the family Bignoniaceae Juss. in Nigeria. Afr J Agric Res.

[CR42] World Health Organization (1985) Chronicle 39:51

[CR43] World Health Organization Centre for Health Development (2002) Traditional medicine: planning for cost-effective traditional health services in the new century—a discussion paper

[CR44] Zirihi GN, Mambu L, Guede-Guina F, Bodo B, Grellier P (2005). In vitro antiplasmodial activity and cytotoxicity of 33 West African plants used for treatment of malaria. J Ethnopharmacol.

